# Genistein-mediated inhibition of glycosaminoglycan synthesis, which corrects storage in cells of patients suffering from mucopolysaccharidoses, acts by influencing an epidermal growth factor-dependent pathway

**DOI:** 10.1186/1423-0127-16-26

**Published:** 2009-03-02

**Authors:** Joanna Jakóbkiewicz-Banecka, Ewa Piotrowska, Magdalena Narajczyk, Sylwia Barańska, Grzegorz Węgrzyn

**Affiliations:** 1 Department of Molecular Biology, University of Gdańsk, Kładki 24, 80-822 Gdańsk, Poland

## Abstract

**Background:**

Mucopolysaccharidoses (MPS) are inherited metabolic disorders caused by mutations leading to dysfunction of one of enzymes involved in degradation of glycosaminoglycans (GAGs). Due to their impaired degradation, GAGs accumulate in cells of patients, which results in dysfunction of tissues and organs. Substrate reduction therapy is one of potential treatment of these diseases. It was demonstrated previously that genistein (4', 5, 7-trihydroxyisoflavone) inhibits synthesis and reduces levels of GAGs in cultures of fibroblasts of MPS patients. Recent pilot clinical study indicated that such a therapy may be effective in MPS III (Sanfilippo syndrome).

**Methods:**

To learn on details of the molecular mechanism of genistein-mediated inhibition of GAG synthesis, efficiency of this process was studied by measuring of incorporation of labeled sulfate, storage of GAGs in lysosomes was estimated by using electron microscopic techniques, and efficiency of phosphorylation of epidermal growth factor (EGF) receptor was determined by using an ELISA-based assay with fluorogenic substrates.

**Results:**

Effects of genistein on inhibition of GAG synthesis and accumulation in fibroblasts from patients suffering from various MPS types were abolished in the presence of an excess of EGF, and were partially reversed by an increased concentration of genistein. No such effects were observed when an excess of 17β-estradiol was used instead of EGF. Moreover, EGF-mediated stimulation of phsophorylation of the EGF receptor was impaired in the presence of genistein in both wild-type and MPS fibroblasts.

**Conclusion:**

The results presented in this report indicate that the mechanism of genistein-mediated inhibition of GAG synthesis operates through epidermal growth factor (EGF)-dependent pathway.

## Background

Mucopolysaccharidoses (MPS) are inherited metabolic disorders, from the group of lysosomal storage diseases, caused by mutations leading to dysfunction of one of enzymes involved in degradation of glycosaminoglycans (GAGs) (for reviews, see [1,2]). Due to their impaired degradation, GAGs accumulate in cells of patients, which results in dysfunction of tissues and organs, including the heart, respiratory system, bones, joints and, in some cases, central nervous system (CNS). The symptoms differ between already described eleven types and subtypes of MPS, which are distinguished biochemically on the basis of the kind of accumulated GAG and the lacking enzyme. Irrespective of the MPS type, the disease is usually fatal, with average expected life span of one or two decades, though severity and clinical progression of MPS vary significantly between patients. These parameters are hardly predictable, even when biochemical and genetic data is available [3], though recent studies revealed a significant progress in this matter [4,5].

Until recently, no effective, approved treatment was available for any MPS type. However, enzyme replacement therapy (ERT), based on an intravenous infusion of an active, recombinant form of a deficient enzyme, is currently used for treatment of patients suffering from MPS I, MPS II and MPS VI [6-10]. This therapy is effective in treatment of somatic symptoms, however, neurological symptoms developed due to GAGs accumulation in CNS cannot be managed by ERT owing to an inefficient delivery of proteins through the blood-brain barrier (for reviews, see [2,11]). The CNS dysfunction-related symptoms occur in some MPS I patients (subtype MPS IH), most of MPS II and MPS VII patients, and all MPS III patients [1], where they are especially severe.

Substrate reduction therapy is one of possible treatments that could be effective in management of CNS-related symptoms of MPS. If a small molecule, capable of crossing the blood-brain barrier, was able to impair GAG synthesis, it could be potentially employed to restore a balance between GAG synthesis and degradation, which is destroyed in cells of MPS patients due to inefficient breaking down of GAGs.

Rhodamine B, a small molecule causing inhibition of GAG synthesis by an unknown mechanism, appeared to be effective in reduction of GAG storage in somatic tissues and in brain of MPS IIIA mice treated at the dose of 1 mg/kg [12]. Moreover, improved behavior has been observed in such mice [13]. On the other hand, the use of rhodamine B in clinical practice is perhaps unlikely due to its possible toxic effects.

Another inhibitor of GAG synthesis is genistein, (4', 5, 7-trihydroxyisoflavone or 5, 7-dihydroxy-3- (4-hydroxyphenyl)-4*H*-1-benzopyran-4-one), a compound from the group of isoflavones. A decreased production of GAGs was observed in fibroblasts of MPS I, MPS II, MPS IIIA and MPS IIIB patients, and a decrease in lysosomal GAG storage was noted after incubation of the cells with genistein at concentrations between 10 and 30 μM [14]. Furthermore, recent pilot clinical studies indicated that treatment of patients suffering from MPS IIIA and MPS IIIB with a genistein-rich isoflavone extract at the dose corresponding to the amount of genistein equal to 5 mg/kg/day resulted in statistically important improvement of all tested parameters, including cognitive functions [15].

The results of the pilot clinical studies [15], as well as of other studies on substrate reduction therapy in lysosomal storage diseases, are encouraging [16]. However, to fully explore a possibility of using genistein as a medicine for treatment of MPS patients, the mechanism of its action must be deciphered. The rationale of the use of genistein in MPS was based on previous observations that maximum synthesis of some GAGs requires either follicle-stimulating hormone or epidermal growth factor (EGF) [17,18]. EGF influences expression of certain genes by binding to its transmembrane receptor that upon interaction becomes an active protein kinase, initiating a specific kinase cascade that finally results in regulation of activity of particular transcription factors [19]. This tyrosine-specific protein kinase activity of the EGF receptor is inhibited by genistein [20,21]. Therefore, it was proposed to call a potential substrate reduction therapy of MPS, which is based on the action of genistein, 'gene-expression targeted isoflavone therapy' (or GET IT) [14]. Nevertheless, this name is still speculative, especially in the light of multiple biological activities of genistein, including its phytoestrogenic properties (for a recent review, see [22]). Therefore, the aim of this work was to elucidate the basic mechanism of genistein-mediated inhibition of GAG synthesis, which results in correction of storage of these compounds in lysosomes of cells from patients suffering from MPS. Since MPS III (Sanfilippo syndrome) is the primary MPS type for which GET IT is being developed, in our studies we focused on this disease, though other MPS types were also considered.

## Methods

### Fibroblasts and cell cultures

Skin fibroblast lines were either initiated from forearm skin biopsies obtained from a healthy volunteer and MPS II, MPS IIIA and MPS IIIB patients (diagnosed biochemically on the basis of determination of urinary GAG levels and measurement of activities of particular enzymes in plasma or leukocytes) or bought from Cascade Biologics (Portland, OR, USA) (adult Human Dermal Fibroblasts; HDFa). Cells were cultured to early confluence in 75 cm^2 ^flasks in Dulbecco's Modiefied Eagle's Medium (DMEM) supplemented with 10% heat inactivated Foetal Bovine Serum (FBS) and Antibiotic Antimycotic Solution (AAS) at 37°C in a humidified atmosphere of 5% (v/v) CO_2_.

### Measurement of GAG synthesis

Fibroblasts were plated in 48-well plates and treated with genistein at concentrations 10 and 30 μM (in DMSO whose final concentration was 0.05%) either alone or in combination with EGF (100 ng/ml) or 17β-estradiol (1 nM) in DMEM supplemented as described above. GAG synthesis was monitored by measuring incorporation of ^35^S (from a radioactive sodium sulfate) into proteoglycans, according to Murata et al. [23]. Cells were labeled with 20 μCi/ml of [^35^S]Na_2_SO_4 _for 24 h in the 50:50 mixture of DMEM and growth medium lacking inorganic sulfate (Minimum Essential Medium Eagle- MEM), supplemented with 2.5% FBS and AAS with appropriate concentrations of additives sustained. After 24 h the fibroblasts were washed six times with PBS and cultures were subjected to papain digestion (0.03% papain in 0.1 M sodium acetate, pH 7.0) [24]. Aliquots of papain digest were used for sulfate incorporation measurements in a scintillation counter. For all experiments, unlabeled control samples were processed in parallel for the determination of DNA concentration. The pellet of fibroblasts obtained after centrifugation of trypsinized cultures was subjected to proteinase digestion, then diluted in TE buffer (12–175 times depending on the intensity of the response to PicoGreen dye) and analyzed according to the protocol provided by the manufacturer of PicoGreen^® ^DNA Quantitation Reagent. GAG synthesis was calculated per DNA amount.

### Electron microscopic analysis of cells

Fibroblasts plated in 12-well plates were treated for 4 days with genistein at concentrations 10 and 30 μM (in DMSO whose final concentration was 0.05%) either alone or in combination with EGF (100 ng/ml) or estradiol (1 nM) in DMEM supplemented with 10% FBS and AAS. Before fixation in 2.5% glutaraldehyde in 0.1 M PBS, the cultures were washed 3 times with PBS. Transmission microscopy studies were performed as described previously [25], using Philips CM100 microscope.

### Estimation of efficiency of phosphorylation of the EGF receptor

Efficiency of phosphorylation of the EGF receptor was estimated by using an ELISA-based assay with fluorogenic substrates to measure phosphorylated EGF receptor in the context of a whole cell (Cell-Based ELISA: Human Phospho-EGF R (Y1068) Immunoassay, R&D Systems Inc., Minneapolis, MN, USA), according to the manufacturer's instruction. A specific and irreversible inhibitor of the EGF receptor, 4- [(3-bromophenyl) amino]-6-acrylamidoquinazoline, abbreviated as PD168393 (purchased from Calbiochem, La Jolla, CA, USA), which acts by covalently modifying cysteine-733, an amino acid located in the catalytic domain of the ATP-binding pocket of the EGF receptor, was used as a control inhibitor. The effect of short-term (30 min) and long-term (24 h) exposure to genistein at 10 and 30 μM or PD168393 at 10 and 30 nM, with or without EGF (100 ng/ml), was examined.

## Results

### Genistein-mediated impairment of GAG synthesis

**Figure 1:**
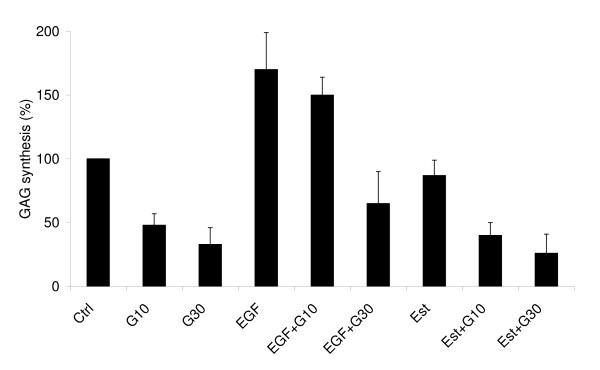
**Effects of genistein, EGF, 17β-estradiol and combinations of genistein with EGF or 17β-estradiol on GAG synthesis in MPS IIIA fibroblasts**. Cells were untreated (Ctrl) or treated with genistein at concentrations 10 or 30 μM (G10 and G30, respectively) alone, EGF alone at 100 ng/ml (EGF), 17β-estradiol alone at 1 nM (Est) or in following combinations: genistein (10 or 30 μM) with EGF (100 ng/ml) (EGF+G10 and EGF+G30, respectively) and genistein (10 or 30 μM) with 17β-estradiol (1 nM) (Est+G10 and Est+G30, respectively). Cell cultures were labeled with 20 μCi/ml of [^35^S]Na_2_SO_4 _for 24 h, and GAG synthesis was monitored by measuring incorporation of ^35^S into proteoglycans. The results were calculated per DNA amount. The results are mean values of three experiments with error bars indicating SD.

As demonstrated previously [14], addition of genistein to cultures of fibroblasts results in impairment of synthesis of GAGs; this was true also for the cell lines used in this study, which derived from MPS IIIA (Fig. 1) and other tested MPS patients (data not shown), and from a non-MPS person (data not shown). As expected, the inhibition of GAG synthesis, measured by incorporation of [^35^S]SO_4_^2-^, revealed the dose-response relationship as the effect was more pronounced at 30 μM genistein than at 10 μM (Fig. 1 and results not shown).

Addition of EGF to the culture of MPS IIIA fibroblasts to final concentration of 100 ng/ml resulted in an increase in efficiency of GAG synthesis relative to untreated cells (Fig. 1). Little decrease in the level of GAG synthesis could be observed by addition of genistein into this experimental system to final concentration 10 μM. In the presence of EGF, the inhibitory effect of genistein could be observed only at 30 μM, although it was significantly less pronounced than in the absence of EGF (Fig. 1). These results indicate that there is an interplay between EGF and genistein in the process of GAG synthesis regulation. Similar results were obtained in experiments with other tested MPS cell lines (data not shown).

No increase in GAG synthesis occurred in MPS IIIA cells in the presence of 1 nM 17β-estradiol, and this compound had also no significant effect on genistein-mediated impairment of GAG production as decreased incorporation of ^35^S was observed even at low (10 μM) concentration of this isoflavone (Fig. 1). This suggests that the estrogen-dependent pathways are not crucial in genistein-mediated inhibition of GAG synthesis. Again, similar results were obtained when other MPS fibroblasts were tested (data not shown).

### Genistein-mediated improvement of GAG storage

**Figure 2:**
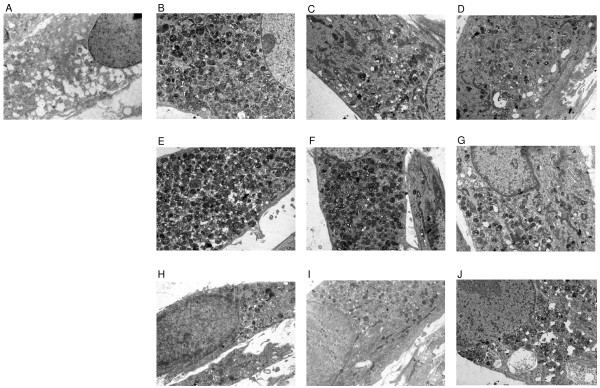
**Effects of genistein, EGF, 17β-estradiol and combinations of genistein with EGF or 17β-estradiol on accumulation of lysosomes containing complex storage structures in MPS IIIAfibroblasts**. Control fibroblasts or MPS IIIA fibroblasts were untreated or treated for 4 days with genistein at concentrations 10 and 30 μM either alone or in combination with EGF (100 ng/ml) or 17β-estradiol (1 nM). Electron micrographs were performed at magnification 1650 x. Panels: (A), control (untreated wild-type cells); (B), untreated MPS IIIA cells; (C), MPS IIIA cells treated with 10 μM genistein; (D), MPS IIIA cells treated with 30 μM genistein; (E), MPS IIIA cells treated with 100 ng/ml EGF; (F), MPS IIIA cells treated with 100 ng/ml EGF and 10 μM genistein; (G), MPS IIIA cells treated with 100 ng/ml EGF and 30 μM genistein; (H), MPS IIIA cells treated with 1 nM 17β-estradiol; (I), MPS IIIA cells treated with 1 nM 17β-estradiol and 10 μM genistein; (J), MPS IIIA cells treated with 1 nM 17β-estradiol and 30 μM genistein.

Effects of storage of GAGs can be visualized by electron microscopic analysis of cultured cells. As expected, evident storage was observed in MPS IIIA fibroblasts, which was not seen in normal cells (control fibroblasts from a healthy person) (Fig. 2A and 2B). In the presence of genistein, the storage was definitely decreased (Fig. 2C and 2D). However, addition of EGF to the MPS IIIA cell culture (to final concentration of 100 ng/ml) resulted in the storage effects even more pronounced than in untreated fibroblasts (Fig. 2E). This enhanced storage resulted in not only a significantly increased number of intracellular lysosomes containing abnormal structures, but also in the presence of some lysosomes with complex inclusions outside cells (Fig. 2E). Supplementation of the cultures with genistein resulted in a partial alleviation of the EGF-mediated effects (Fig. 2F and 2G). Results of experiments with 17β-estradiol (Fig. 2H, I and 2J) were roughly similar to those obtained without any treatment of cell cultures and when genistein was used alone. The electron microscopic analysis could be quantified by counting the number of complex storage structures (Table 1). In summary, the results obtained in electron microscopic studies of fibroblasts cultured in the presence of various compounds are compatible with those obtained in experiments with measurement of GAG synthesis, and support the conclusion that genistein-mediated inhibition of GAG production acts by influencing EGF-dependent process(es), rather than those dependent on estrogen activities.

**Table 1:** Effects of genistein, EGF, 17β-estradiol and combinations of genistein with EGF or 17β-estradiol on accumulation of lysosomes containing complex storage structures in MPS IIIA fibroblasts

Cells	Added agents			^a ^Average number of complex storage structures per 100 μm^2^
		
	Genistein (μM)	EGF (ng/ml)	17β-estradiol (nM)	
Control	0	0	0	2
MPS IIIA	0	0	0	38
MPS IIIA	10	0	0	7
MPS IIIA	30	0	0	5
MPS IIIA	0	100	0	67
MPS IIIA	10	100	0	26
MPS IIIA	30	100	0	11
MPS IIIA	0	0	1	21
MPS IIIA	10	0	1	13
MPS IIIA	30	0	1	8

^a ^Electron micrographs obtained as shown in Fig. 2 were analyzed by counting complex storage structures per 100 μm^2^.

### Effects of genistein on phosphorylation of the EGF receptor

The first step in the EGF-dependent signal transduction pathway is autophosphorylation of the EGF receptor, upon binding of EGF. Therefore, to test whether genistein influences the EGF-mediated pathway, we have estimated the kinase activity of the EGF receptor by measuring efficiency of phosphorylation of this protein in the absence and presence of EGF and/or genistein. Fibroblasts were cultured in the standard medium, which in a short-term experiment was followed by 30 min incubation with (or without) genistein or a specific and irreversible tyrosine kinase inhibitor (PD168393), and then by incubation with 100 ng/ml of EGF for 5 min. In a long-term experiment, fibroblasts were treated with the mixture of genistein and EGF for 24 h.

**Figure 3:**
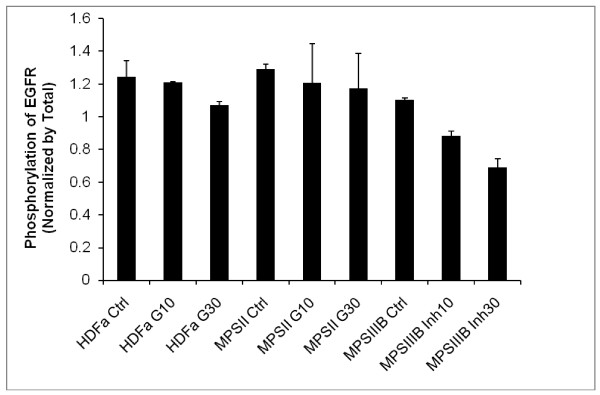
**Short-term effects of EGF and genistein on phosphorylation of the EGF receptor**. Fibroblasts were cultured in the absence of EGF, which indicated the residual phosphorylation level (baseline value), and in the presence of EGF (100 ng/ml for 5 min) without any inhibitor (Ctrl) or with genistein (either 10 or 30 μM, marked as G10 or G30, respectively) or PD168393 (either 10 or 30 nM, marked as Inh10 or Inh30, respectively) added 30 min before EGF. Results of experiments with HDFa (wild-type), MPS II and MPS IIIB cell (fibroblast) lines are shown. Other tested fibroblasts gave similar results (results not shown). The results were calculated as phospho-EGF receptor fluorescence at 600 nm normalized to the total EGF receptor fluorescence at 450 nm. Therefore, the obtained normalized values are in arbitrary units. Values represent mean ± range of duplicate determinations.

**Figure 4:**
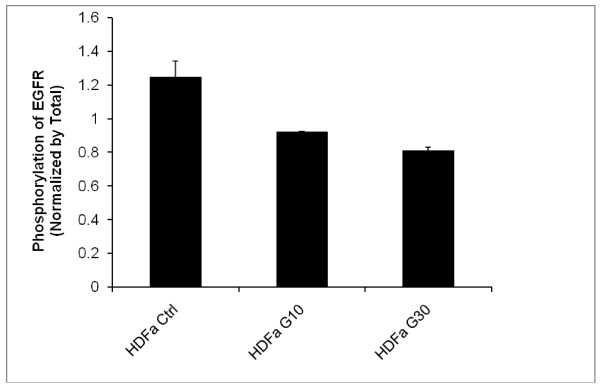
**Long-term effects of EGF and genistein on phosphorylation of the EGF receptor**. Fibroblasts were cultured in the absence of EGF, which indicated the residual phosphorylation level (baseline value), and in the presence of EGF (100 ng/ml for 24 h) (Ctrl) without genistein or with genistein (either 10 or 30 μM, marked as G10 or G30, respectively) added together with EGF. Results of experiments with HDFa fibroblasts (wild-type) are shown. Similar effects were observed in experiments with MPS II, MPS IIIA and MPS IIIB fibroblasts (results not shown). The results were calculated as phospho-EGF receptor fluorescence at 600 nm normalized to the total EGF receptor fluorescence at 450 nm. Therefore, the obtained normalized values are in arbitrary units. Values represent mean ± range of duplicate determinations.

As expected, we observed a significant increase in phosphorylation of the EGF receptor in the presence of 100 ng/ml EGF in the culture of wild-type fibroblasts relative to the non-stimulated culture (Figs. 3 and 4). The same phenomenon (i.e. stimulation of the EGF receptor phosphorylation by EGF) was detected in cultures of other tested fibroblasts, including those obtained from patients suffering from MPS (Fig. 3). The tyrosine kinase inhibitor PD168393, used as a positive control, abolished this phosphorylation stimulation (Fig. 3).

**Figure 5:**
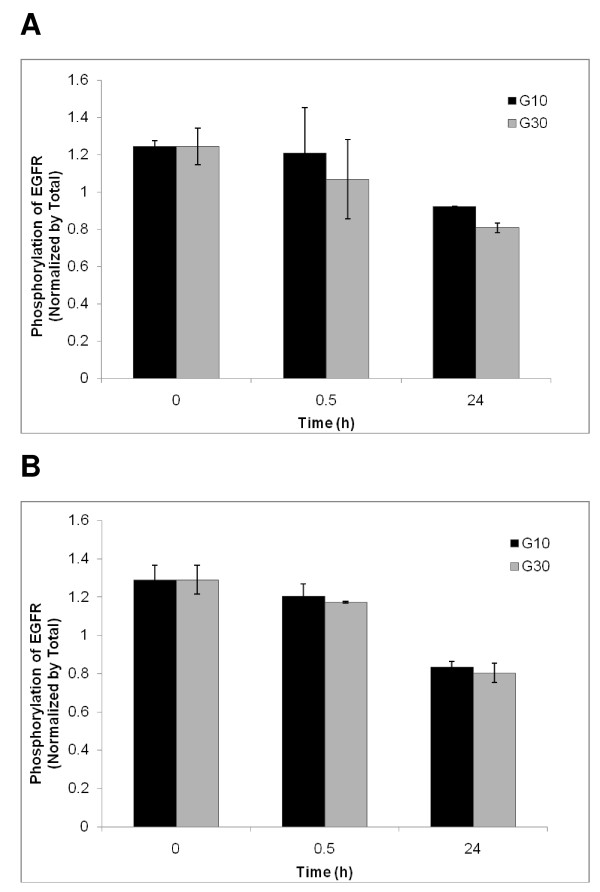
**Effects of the length of incubation of cell cultures with genistein on inhibition of EFG receptor phosphorylation**. Experiments were performed as described in Methods and in captions to Figs. 3 and 4. Genistein was used at final concentrations of 10 μM (black columns) or 30 μM (grey columns). Results of experiments with HDFa (panel A) and MPS II (panel B) fibroblasts are shown. Other tested fibroblasts gave similar results (results not shown). Values represent mean ± range of duplicate determinations.

When fibroblasts were incubated with genistein together with EGF or prior to addition of EGF, the phosphorylation stimulation was reduced significantly, and the effect was better pronounced at higher genistein concentration (30 μM) than at its lower concentration (10 μM). This was true for both experimental systems (short-term and long-term), although the efficiency of phosphorylation inhibition was generally more efficient when genistein was incubated together with EGF for 24 h (Fig. 4) than if genistein was added to the cell culture 30 min prior to addition of EGF and further incubation for 5 min (Fig. 3). The dependence of the phosphorylation efficiency on the length of cell culture incubation with genistein is depicted in Fig. 5, indicating that longer incubation resulted in stronger inhibition of phosphorylation. Therefore, we conclude that genistein affects the EGF-mediated signal transduction pathway.

## Discussion

Although the use of genistein in substrate reduction therapy for mucopolysaccharidoses has been proposed on the basis of inhibition of GAG synthesis and accumulation in fibroblasts of MPS patients [14], and a pilot clinical study revealed positive effects of the treatment with genistein-rich isoflavone extract [15], the mechanism of regulation of GAG synthesis by genistein remained speculative. It was assumed that genistein might regulate expression of genes involved in GAG synthesis, and the therapy was named "gene expression-targeted isoflavone therapy" (GET IT). This assumption was based on previous findings, which demonstrated an increased GAG synthesis in the presence of EGF and inhibition of the kinase activity of the EGF receptor by genistein [17,18,20]. Nevertheless, since there are many biological activities of genistein [22], this hypothesis had to be tested.

Results presented in this report confirmed that an excess of EGF stimulates GAG synthesis, which is also true in MPS fibroblasts, and indicated that under such conditions accumulation of GAGs is more efficient than in untreated patients' cells. In the latter case, the storage of abnormal structures was so efficient that some lysosomes containing complex undigested structures appeared outside cells. The effects of EGF on GAG synthesis and storage could be partially reversed only at relatively high concentration of genistein (30 μM). Moreover, genistein reduced EGF-mediated stimulation of autophosphorylation of the EGF receptor, the first step in the EGF-dependent signal transduction pathway. These results strongly suggest that effects of genistein on GAG synthesis and accumulation in MPS cells is due to inhibition of the kinase activity of the EGF receptor and subsequent effects on expression of particular genes that are regulated through EGF-dependent signal transduction. Therefore, it appears that the name "gene expression-targeted isoflavone therapy" (GET IT) is valid, indeed. Although genistein is a phytoestrogen, it seems that this activity is not important in the process of regulation of GAG synthesis by this isoflavone as no significant effects on this process were observed in the presence of 17β-estradiol.

Despite a likely regulation of GAG synthesis by genistein through negative control of EGF-dependent signal transduction and expression of certain genes, we cannot exclude other biological effects of this isoflavone, which might contribute to observed positive effects during clinical studies [15]. One of examples is an antioxidant activity of genistein [26]. In this light, recent results which signaled an involvement of the reactive oxygen species in the genesis of neurodegeneration in MPS IIIB [27], may suggest that genistein could have positive effects on neurological and cognitive functions of MPS III patients (observed in the clinical study [15]) also as an antioxidant. Thus, although the primary mechanism of therapeutic effects of genistein for MPS III patients appears to be modulation of the EGF-dependent signal transduction, other genistein-mediated processes should also be considered and investigated. Nevertheless, because of the encouraging results of the pilot clinical study [15,16], no significant side effects reported [15] and a good safety profile of the use of genistein (revised and discussed recently [28]), the gene expression-targeted isoflavone therapy (GET IT) remains a hopeful potential treatment of patients suffering from MPS III and perhaps also some other MPS types.

## Conclusion

In this report we demonstrate that the mechanism of genistein-mediated inhibition of GAG synthesis operates through epidermal growth factor (EGF)-dependent pathway. Therefore, since this pathway is involved in regulation of gene expression, the therapy of mucopolysaccharidosis based on this reaction can be called 'Gene Expression-Targeted Isoflavone Therapy' (GET IT).

## Abbreviations

CNS: central nervous system; EGF: epidermal growth factor; GAG: glycosaminoglycan; GET IT: gene expression-targeted isoflavone therapy; MPS: mucopolysaccharidosis.

## Competing interests

The authors declare that they have no competing interests.

## Authors' contributions

JJ-B designed experiments and participated in data interpretation and in preparing a draft of the manuscript. EP performed biochemical experiments and participated in data analysis and in preparing the manuscript. MN and SB performed electron microscopic studies. GW supervised the design and execution of the study, performed the final data analyses and contributed to the writing of the manuscript.
